# Replantation of an Avulsed Maxillary Incisor after 12 Hours: Three-Year Follow-Up

**Published:** 2013-01-20

**Authors:** Hamid Moradian, Samaneh Badakhsh, Mehran Rahimi, Somayeh Hekmatfar

**Affiliations:** 1 Department of Pediatric Dentistry, Shiraz University of Medical Sciences, Shiraz, Iran; 2 Department of Pediatric Dentistry, Zanjan University Of Medical Sciences, Zanjan, Iran; 3 Department of Pediatric Dentistry, Ardabil University of Medical Sciences, Ardabil, Iran

**Keywords:** Media, Milk, Replantation, Tooth Avulsion, Trauma

## Abstract

Tooth avulsion is defined as the complete displacement of the tooth out of its alveolar socket. The treatment of choice is immediate replantation or if that is not possible, placement of the tooth in an appropriate storage media. This report presents replantation of an avulsed maxillary central incisor after 12 hours of storage in milk. The tooth was replanted and splinted. One week later, it was treated endodontically and calcium hydroxide dressing was placed for 1 month; subsequently, the tooth was obturated with gutta-percha. During three years of follow-up, no evidence of ankylosis or inflammatory resorption was observed. After three years, the tooth was stable and remained functional and esthetically acceptable.

## 1. Introduction

Dental trauma may result in many significantly undesirable functional, esthetics and even psychological effects. The main causative factors for the trauma are fights and sports [1] .

Tooth avulsion (exarticulation) is characterized as total displacement of the tooth out of its alveolar socket with damage to the periodontal ligament, cementum, alveolar bone, as well as gingival and pulpal tissues [2, 3]. Its prevalence in permanent dentition is about 1% to 16% of traumatic injuries [4, 5]. Avulsion of the permanent teeth occurs more frequently in children between 7 to 9 years in which the periodontal ligament structure is still loose and the surrounding bone of the newly erupted teeth is slightly mineralized. Thus, there is only a minimal resistance to the extrusive forces. Maxillary central incisors are more prone to avulsion [4, 6].

The most prevalent outcomes of avulsion are pulp necrosis and root resorption [7,8]. In an avulsed tooth, the neurovascular supply is severely damaged, so pulp necrosis is predictable. In case of damage to protective structures of the root surface (i.e. cementum, periodontal ligament), a chemotactic process will attract clastic cells to site of injury, initiating the resorption process. External inflammatory resorption is a progressive inflammatory process and a necrotic pulp can induce and sustain this resorptive destruction. Clinically, the tooth does not respond to vitality tests; tooth crown discoloration may develop, and the tooth may be tender to percussion. Periapical radiolucencies associated with resorption are often seen. The other type of resorption which occurs due to the lack of the vital periodontal ligament is called replacement resorption (ankylosis). In this type of resorption, while osteoclastic activity is resorbing the root surface, osteoblastic activity creates the new bone. Clinically, the tooth is immobile and presents a metallic sound to percussion. Infraoccluded teeth are the common observation. There may be no radiographic signs [8]. Ample evidences have shown that there is a high correlation between patients’ age at the time of injury and resorption rate, as well as tooth’s infraposition level. In fact, when ankylosis occurs before the growth spurt of the patient, an infraoccluded tooth is commonly seen. Thus, in this situations long term treatment planning is needed. However, after growth spurt the rate of the resorption is slower and the tooth may be preserved for a longer period [8, 9].

Avulsion causes complete rupture in the periodontal ligament (PDL) tissue; a part remains on the alveolar socket walls and other PDL remnants are seen attached to the root surface of the avulsed tooth. The part of the PDL attached to the alveolar wall will preserve its vitality, so there is no need for additional treatment, while the portion attached to the root surface may be at risk of necrosis. The most important factor in determining the final prognosis of an avulsed tooth is the presence of viable periodontal ligament cells on the root surface [10]. Immediate replantation within 20-30 minutes after the injury is considered as the treatment of choice for tooth survival [10]. In some conditions due to parents' anxiety, lack of knowledge or non-cooperating patient where immediate replantation is not feasible, the tooth should be kept in an appropriate storage media [11]. Regardless of the length of exarticulation time and the transporting condition, replantation of avulsed teeth should be carried out [12].

This article reports the management of an avulsed maxillary permanent incisor that had been replanted after 12 hours and describes the successful clinical and radiographic findings observed after 3 years of follow-up.

## 2. Case Report

A 12 year old boy was referred to the pediatric department of Shiraz University of Medical Sciences (Shiraz, Iran) for the treatment of his traumatized teeth. The accident occurred 12 hours before his referral, due to a fall.

The patient seemed healthy with no sign and symptom of cerebral involvement (i.e. Amnesia, headache, unconsciousness, vomiting, dizziness, visual disturbances, and cognitive impairments) [13]. Extra-oral examination showed no swelling, soft tissue injuries, asymmetry or even pain.

**Figure 1.**
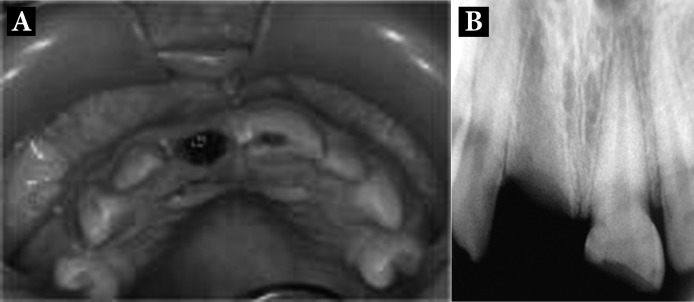
A) Intraoral examination; B) Radiographic examination

Intra-oral examination demonstrated a permanent dentition, with class I skeletal and dental relationship; the oral hygiene was good. Clinically, dental traumas included complicated crown fracture of the maxillary left central incisor (tooth 21) and avulsion of the maxillary right central incisor (tooth 11) (Figure 1A). The time that had elapsed between the fall and his referral was 12 hours and the tooth had been stored in milk during this period. The avulsed tooth had as closed apex with moist periodontal tissue on its root surface and no additional damages were found.

To complete the examination, periapical radiographs were taken. Radiographs showed a normal socket for tooth 11 and non-fractured root for tooth 21 were observed (Figure 1B).

The parents were informed about the treatment procedure and the expected prognosis. The treatment consisted of immediate replantation of avulsed tooth and partial pulpotomy of tooth 21 using hard setting calcium hydroxide.

The avulsed tooth was soaked in 2% sodium fluoride gel and local anesthesia was administered with lidocaine 2% with 1:100000 epinephrine. First, the alveolar socket was prepared for proper positioning of the avulsed tooth. Then the tooth was rinsed with normal saline and carefully repositioned so that no periodontal ligament was damaged. Finally, we splinted the tooth with round wire (0.028 inch) and light cure resin composite. Another periapical radiograph was taken to confirm correct tooth repositioning. The splint was left in place for 2 weeks.

Based on medical history, the patient did not need any anti tetanus booster. Systemic Tetracycline 250 mg and 0.2% chlorhexidine mouth rinse were prescribed for 7 days.

One week later, tooth 11 was treated endodontically and calcium hydroxide dressing was placed for 1 month.

**Figure 2.**
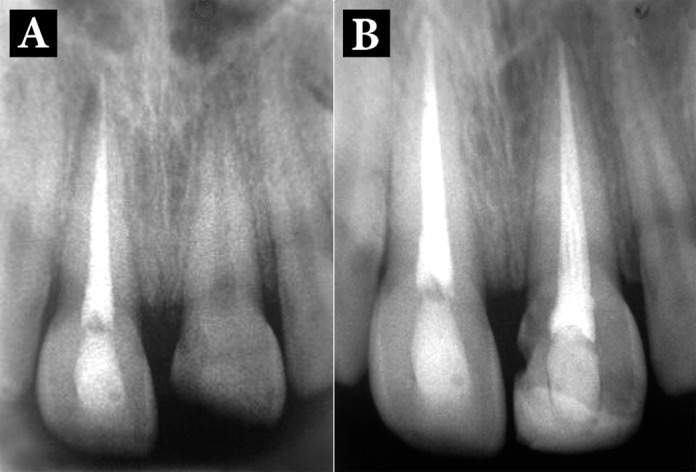
Follow-up radiographic images; A) One month; B) One year

Subsequently, the tooth was obturated with gutta-percha and restored with composite resin (Figure 2A). The patient was seen postoperatively at 6 and 12 weeks after replantation, and then followed-up every six months. In the first follow-up, it was found that tooth 21 has lost its vitality; therefore, the patient was referred for root canal therapy (Figure 2B).

**Figure 3.**
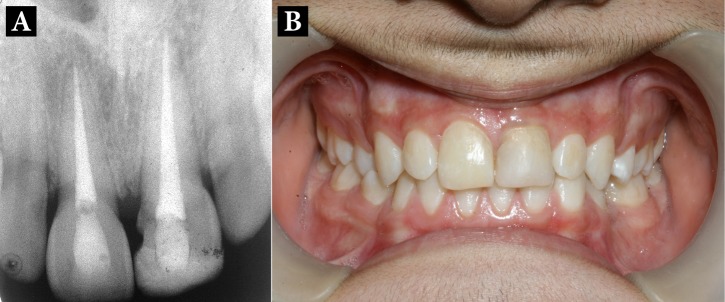
Three-year follow up images; A) Radiographic; B) Intraoral

The patient was followed-up for three years. The tooth remained functional and was esthetically acceptable. The percussion tone and the tooth mobility were normal. Radiographically, no inflammatory or replacement root resorption was detected and the adjacent anterior teeth remained asymptomatic (Figure 3A, B).

## 3. Discussion

Treatment of avulsion is often challenging and should be managed as an emergency. The golden time for replantation is 20-30 minutes; if it is not possible, the tooth should be kept in an appropriate storage media for preserving the viability of the periodontal ligament cells. The most preferable storage medias are Viaspan and HBSS (Hank’s Balanced Salt Solution) [12]. Viaspan can preserve the cell viability even for 96 hours [14]. Unfortunately, these media are not accessible at the site of trauma. Milk has sufficient nutrients, appropriate PH and osmolality with fewer bacteria. Contact lens saline may also be used if available. Placing an avulsed tooth in milk at room temperature can preserve the cell viability for up to 6 hours [12]; in the present case report, keeping the tooth for 12 hours showed a good prolonged prognosis. The extra-alveolar period and the type of transporting condition are not necessarily definite contra-indications for replantation. A study by Shiu-yin Cho demonstrated that replantation of the tooth which was left dry for 18 hours before replantation had acceptable functional and esthetical prognosis during 2 years of follow-up [6]. Khalilak et al. reported a case which replantation was done after 270 min. During 5 years follow-up, the tooth showed signs of ankylosis (infraocclusion); however, still it was stable [11].

If the physiologic media (e.g. cold milk or contact lens saline solution) are not available, soaking the tooth in non-physiologic media such as saliva (placing the tooth in buccal vestibule), normal saline and tap water is a superior option compared to keeping it in a dry condition. Although they are detrimental to the periodontal cells, saliva and saline can maintain the cell viability for 2 hours and tap water can preserve them for 20 minutes [12].

Another tissue which may be damaged during avulsion is the pulpal tissue. In closed apex teeth with narrow apical foramen, prophylactic endodontic treatment should be performed after replantation. But in wide open apex teeth, as there is a chance for revascularization, accurate observation is recommended. In these teeth, uninfected necrotic pulp may act as scaffold for later regenerative processes. Kling et al. reported that there is more chance of revascularization, if the tooth is replanted within 45 minutes [15]. Therefore, the open apex teeth should be controlled radiographically; in case of any signs of necrosis such as root resorption, root canal therapy is the treatment of choice [7, 16, 17]. In this case report, the avulsed tooth had a mature root, thus one week after the replantation, the pulp was extirpated expired.

Ankylosis (replacement resorption) is defined as a fusion of the alveolar bone and root surface. It seems that the absence of viable periodontal ligament cells is the major etiological factor. During ankylosis, the tooth root merges with the remodeled normal bone and is gradually replaced with bone. The latter can be recognized within 4-8 weeks after replantation. Clinically, the tooth is immobile and might be infraoccluded and have a metallic percussion sound. Radiographically, the periodontal space disappears [8, 18].

In the present case, although the tooth was stored in milk for the whole extra-alveolar duration, some parts of the periodontal ligament tissues seemed to have become necrotic because of the prolonged extra-alveolar period., The authors chose to soak the avulsed tooth in 2% sodium fluoride gel for 20 minutes in order to reduce ankylosis progression; however, this procedure is not standard protocol. Fortunately, during the three years of follow-up ankylosis was not evident clinically or radiographically.

It is important to highlight that avulsion is a severe dental injury; if it is managed immediately and appropriately, the avulsed teeth can be preserved and be functional for some years. The three year follow-up indicated success of this treatment protocol.

## 4. Conclusion

Although acceptable results were observed in the three-year follow-ups, further observation is required. Moreover, further clinical and in vivo studies are recommended to confirm that replantation after extended extra-oral time is applicable clinically and that fluoride application contributes to success.
